# Systems Biology Analysis Merging Phenotype, Metabolomic and Genomic Data Identifies *Non-SMC Condensin I Complex*, *Subunit G (NCAPG)* and Cellular Maintenance Processes as Major Contributors to Genetic Variability in Bovine Feed Efficiency

**DOI:** 10.1371/journal.pone.0124574

**Published:** 2015-04-15

**Authors:** Philipp Widmann, Antonio Reverter, Rosemarie Weikard, Karsten Suhre, Harald M. Hammon, Elke Albrecht, Christa Kuehn

**Affiliations:** 1 Leibniz Institute for Farm Animal Biology, Institute for Genome Biology, Genome Physiology Unit, Dummerstorf, Germany; 2 Leibniz Institute for Farm Animal Biology, Institute for Nutritional Physiology “Oskar Kellner”, Dummerstorf, Germany; 3 Leibniz Institute for Farm Animal Biology, Institute for Muscle Biology and Growth, Dummerstorf, Germany; 4 CSIRO Agriculture Flagship, Brisbane, Australia; 5 Weill Cornell Medical College in Qatar, Doha, State of Qatar; 6 Institute of Bioinformatics and Systems Biology, Helmholtz Zentrum München, German Research Center for Environmental Health, Neuherberg, Germany; 7 Faculty of Agricultural and Environmental Sciences, University Rostock, Rostock, Germany; University of Lleida, SPAIN

## Abstract

Feed efficiency is a paramount factor for livestock economy. Previous studies had indicated a substantial heritability of several feed efficiency traits. In our study, we investigated the genetic background of residual feed intake, a commonly used parameter of feed efficiency, in a cattle resource population generated from crossing dairy and beef cattle. Starting from a whole genome association analysis, we subsequently performed combined phenotype-metabolome-genome analysis taking a systems biology approach by inferring gene networks based on partial correlation and information theory approaches. Our data about biological processes enriched with genes from the feed efficiency network suggest that genetic variation in feed efficiency is driven by genetic modulation of basic processes relevant to general cellular functions. When looking at the predicted upstream regulators from the feed efficiency network, the *Tumor Protein P53* (*TP53*) and *Transforming Growth Factor beta 1* (*TGFB1*) genes stood out regarding significance of overlap and number of target molecules in the data set. These results further support the hypothesis that *TP53* is a major upstream regulator for genetic variation of feed efficiency. Furthermore, our data revealed a significant effect of both, the *Non-SMC Condensin I Complex*, *Subunit G (NCAPG) I442M* (rs109570900) and the *Growth /differentiation factor 8 (GDF8) Q204X* (rs110344317) loci, on residual feed intake and feed conversion. For both loci, the growth promoting allele at the onset of puberty was associated with a negative, but favorable effect on residual feed intake. The elevated energy demand for increased growth triggered by the *NCAPG 442M* allele is obviously not fully compensated for by an increased efficiency in converting feed into body tissue. As a consequence, the individuals carrying the *NCAPG 442M* allele had an additional demand for energy uptake that is reflected by the association of the allele with increased daily energy intake as observed in our study.

## Introduction

Recent studies highlighted the *Non-SMC Condensin I Complex*, *Subunit G (NCAPG)/ Ligand Dependent Nuclear Receptor Corepressor-Like (LCORL)* region in several mammalian genomes including human and cattle being associated with genetic modulation of growth [1–8]. Specifically, the *NCAPG I442M* (*rs109570900*) locus had been associated with pre- and postnatal growth in cattle [1,9,10]. Furthermore, it had been demonstrated that the highest effects of this genetic variant are expressed at the onset of puberty, which is suggested as the inflection point of the growth curve [11]. A potential mechanism explaining the physiological background of the divergent growth associated with *NCAPG I442M* is an elevated plasma arginine level [8]. Network analyses added information on key genes interacting with *NCAPG I442M* for the modulation of growth at the onset of puberty [12]. Effects of *NCAPG I442M* on feed efficiency at the onset of puberty, however, were not yet evaluated, although recently the corresponding chromosomal region was associated with genetic variation of feed intake [13].

Feed costs form a large proportion of total costs for production in livestock, and including information about feed efficiency in selection decisions would therefore improve the profitability and environmental impact of livestock production. A traditional parameter for feed efficiency in livestock is the ratio of feed intake to body weight gain defined as feed conversion ratio (FCR). However, FCR is not recommended as a target trait for the improvement of efficiency in production systems as it is highly correlated with growth rates and therefore might result in increased maintenance costs of mature animals [14]. An alternative to measure feed efficiency is residual feed intake (RFI), which was introduced by Koch et al. [15] and is the difference between actual feed intake and predicted feed intake after accounting for the requirements for production and maintenance. For RFI, low to moderate heritabilities (0.16–0.43) had been described in beef cattle [16–18]. In contrast to FCR, RFI had been described as phenotypically and genetically independent of size and growth rate [18]. In spite of this overall independence between RFI and growth, single loci might nevertheless impact both traits either on the same or opposite directions. Differences of correlated effects for single genes compared to genetic correlations reflecting the action of the whole genome have been demonstrated, e.g., for *DGAT1 K232A (rs109234250)* regarding milk fat and milk protein yield [19–21].

The physiological basis for variation in RFI was investigated by Herd and Arthur [22], who highlighted major physiological processes likely contributing to RFI (i.e. protein turnover, tissue metabolism and stress, digestibility, heat increment and fermentation; physical activity, body composition, and feeding patterns). From these results it can be concluded that feed efficiency is a complex trait controlled by a wide range of biological processes and very likely regulated by a large number of different genes. Genome wide association studies (GWAS) support this assumption by identifying QTL and candidate genes for feed efficiency on nearly every cattle chromosome [23–30]. Gene expression experiments further identified candidate genes and functional processes related to RFI [31].

Recent studies and literature showed that systems biology approaches are powerful in the dissection of complex traits [32–34]. Systems biology aims at obtaining a comprehensive view of the dynamics within systems by combining data from different levels of knowledge in one approach [35]. In contrast to single-locus GWAS, systems biology approaches are capable of pointing out gene-gene interactions that can be visualized in network structures. Approaches combining genetic with metabolomic data are valuable for the detection of genes and pathways that are involved in the manifestation of complex traits [36–38]. Thus, by combining physiological and genetic information from a number of traits that contribute to variation in feed efficiency, a systems biology approach should be capable to reveal new insights into the genetic modulation of feed efficiency.

As previously mentioned, metabolic processes have a major impact on feed efficiency [39]. Furthermore, a previous study had suggested a substantial impact of the *NCAPG I442M* locus on specific metabolites [8]. Thus, the aim of our study was to evaluate if there was variation in feed efficiency at the onset of puberty in a cattle cross population that segregated for the growth-associated *NCAPG I442M* locus [8,40] and if *NCAPG I442M* indeed had an effect on feed efficiency traits at this ontogenetic stage. The available data would add further details to the potential functional role of the *NCAPG I442M* locus for body mass accretion. For comparison, an analogous analysis was conducted for *GDF8 Q204X* (rs110344317), a mutation in a well-known major gene affecting mammalian growth [41–45]. Finally, our study also evaluated key mechanisms of divergent feed efficiency at the onset of puberty applying a systems biology network approach, which merges phenotypic, genomic and metabolomics data.

## Materials and Methods

### Animals, housing conditions and blood sampling

All experimental procedures were carried out according to the German animal care guidelines and were approved and supervised by the relevant authorities (Landesamt für Landwirtschaft, Lebensmittelsicherheit und Fischerei Mecklenburg-Vorpommern, Rostock; Landkreis Bad Doberan, Bad Doberan, Germany) of the State Mecklenburg-Vorpommern, Germany.

The present study included data from 237 male F_2_ individuals from a Charolais x German Holstein cattle resource population (SEGFAM) [40]. The animals were generated via superovulation and subsequent embryo transfer to virgin German Holstein heifers. Environmental, feeding and housing conditions were standardized due to uniform environment in the experimental unit of the Leibniz Institute for Farm Animal Biology (FBN), Dummerstorf, Germany. Immediately after birth, the calves were removed from the mother and fed a milk replacer diet. Calves were weaned at day 121 and fed a hay and concentrate diet *ad libitum*. Hay contained 11% crude protein and 32% crude fiber, and the concentrate was a mixture of barley, molasses chips, soybean extraction meal, molasses, minerals and a vitamin premix resulting in a composition of 15% crude protein, 9% crude fiber, 2% crude fat, 0.8% calcium, 0.3% phosphorus and 0.3% sodium (RM 2007, Vollkraft Mischfutterwerke GmbH, Rendsburg, Germany). Energy contents were 9.0 MJ ME/kg dry matter for hay and 11.3 MJ ME/kg dry matter for the concentrate. The offered proportion of hay to concentrate was 1:3. The animals were kept in a tight stall barn, which enabled separate recording of the daily consumed feed for each animal. Body weight was monthly documented.

Blood sampling of animals for the metabolic analyses was carried out as previously described [8]. Briefly, the animals were blood sampled by a standardized procedure at 240 days of age at 7:30 AM after a fasting period of 12 hours. Blood was collected in EDTA tubes (Sarstedt AG & Co, Nümbrecht, Germany) and immediately stored on ice to interrupt enzyme activities and further processing of metabolites. Within 30 min, plasma was obtained from these samples by centrifugation. Subsequently, plasma samples were stored at -80°C until they were used for the metabolomic analyses. For genotyping, blood samples were obtained at slaughter and leukocytes were extracted and stored at -20°C until DNA isolation.

### Determination of phenotypes

#### RFI, FCR and daily energy intake (dEI)

RFI, FCR and dEI were investigated from six to nine months of age. The time interval was selected because it corresponds to the onset of puberty in the SEGFAM population [8], and we expected parameters affecting feed efficiency to become distinct in this interval due to the significant changes in metabolic and physiological parameters occurring during puberty. Furthermore, previous studies showed that the *NCAPG* I442M locus was predominantly associated with growth and metabolic traits in the SEGFAM population at this time point [1,8], which raised the question for an additional impact also on energy utilization or feed efficiency. Finally, the metabolomic data obtained at 240 days of age fit the respective time interval of the onset of puberty [46], thus enabling the analysis of RFI, FCR, dEI and the metabolomic data obtained at the same time point.

RFI was calculated by modeling the animals’ energy intake considering the average daily gain and metabolic mid-weight (i.e., mid body weight from month 6 to 9 raised to the power of 0.75). The following statistical model was fitted:

$$dEI_{i}=\beta_{0}+\beta_{1}ADG_{i}+\beta_{2}MMWT_{i}+e_{i}$$

where dEI is the average daily energy intake of animal *i* in Megajoule metabolizable energy (MJ ME) from month 6 to 9, β_0_ is the intercept, β_1_ is the regression coefficient of feed intake on average daily gain of animal *i* from month 6 to 9 (ADG), β_2_ is the regression coefficient of feed intake on metabolic mid-weight of animal *i* from month 6 to 9 (MMWT_*i*_), and e_*i*_ is the residual error term which represented the RFI for animal *i*. dEI in MJ ME for each animal was determined from the amount and the energy content of consumed feed within the test period. ADG_*i*_ was calculated as the total body weight gain within the test period divided by the number of days in this period. MMWT was calculated as the initial body weight (body weight at month 6) plus one-half of the body weight gain during the test period raised to the power of 0.75 [47–49]. The above regression model is widely used for the calculation of RFI [17,50]. Based on the outcome of the model, the individuals can be subdivided into efficient animals (individuals with lower feed intake than predicted) and inefficient animals (individuals with higher feed intake than predicted) with efficient animals characterized by a negative RFI value and inefficient animals characterized by a positive RFI value.

FCR is another parameter for feed efficiency that had been used in the past. In the present study, FCR was calculated for each animal as ratio of its energy intake in MJ ME from month 6 to 9 and its weight gain in the period from month 6 to 9 (energy intake_6-9_/weight gain_6-9_).

#### Metabolites

Our systems biology analysis of feed efficiency is complemented by quantitative measurements of plasma metabolites including acylcarnitines, amino acids, lysophosphatidylcholines, phosphatidylcholines, sphingomyelins, biogenic amines and sugars from metabolomics profiling of the animals. Metabolomic analyses were conducted as described by Römisch-Margl et al. [51] and Weikard et al. [8]. Briefly, from each plasma sample a total of 221 known metabolites (see Weikard et al. [8] for details) comprising 48 acylcarnitines, 18 amino acids, 9 lysophosphatidylcholines, 70 phosphatidylcholines, 16 sphingomyelins, 8 biogenic amines and 52 sugars were extracted using the Biocrates targeted metabolomics technology (http://www.biocrates.com/) followed by quantifying each metabolite via electrospray ionization tandem mass spectrometry (ESI-MS/MS). This method fulfills FDA-Guideline requirements (U.S. Department of Health and Human Services 2001) which imply the proof of reproducibility. In what follows, the metabolite nomenclature is adapted to the Lipid maps classification system [52], where an abbreviation denoted as C*x*:*y* describes the number of carbon atoms (*x*) and the number of double bonds (*y*) in lipid C. Further, *a* and *e* in phosphocholine molecules will denote acyl and ether side chains, respectively.

### Genotyping

The animals were investigated for their *NCAPG I442M* (*rs109570900*) and *GDF Q204X* (*rs110344317*) genotypes as described by Weikard et al. [8]. For further GWAS and network analyses, a subset of 176 animals was genotyped with Illumina Bovine SNP50 v2 (50k) chips, which were processed according to Illumina Infinium HD Assay Ultra guidelines and read out on an Illumina iScan system. Quality control with Illumina Genome Studio v2011 was carried out as follows: all SNP clusters with either a call frequency < 0.98, a GenTrain Score < 0.68 or a Chi^2^-test for deviation from Hardy-Weinberg equilibrium < 0.005 were manually checked and re-clustered if possible. After manual re-clustering, only autosomal SNPs with a call frequency > 0.85 and a minor allele frequency > 0.01 (n = 44,507 SNPs passing filtering) as well as all samples with a call rate > 0.98 were included in further analyses.

### Data analysis

#### *NCAPG* and *GDF8* association analyses and GWAS

Association analyses were performed with the software Qxpak v5.05 [53] on a Unix operating system. First, targeted associations between the putatively functional loci *NCAPG I442M* and *GDF8 Q204X*, respectively, and the traits RFI, FCR, dEI, daily weight gain from six to nine months of age (ADG), plasma arginine and carnitine (C0) concentration were calculated. In the next step, a GWAS with untargeted SNPs based on the 50k Illumina bead chip were performed for RFI, FCR, dEI and all 221 metabolites. Qxpak determines statistical significances (p-values) for each SNP-trait combination via a likelihood ratio test. The following mixed model for each SNP was fitted in Qxpak:

$$y_{ik}=X_{i}\varphi +Z_{k}g+u_{i}+e_{ik}$$

where *y*_*i*_ contains the phenotypic records of animal *i*, *X*_*i*_ is the *i*th row of an incidence matrix of fixed effects, *φ* contains the fixed effects solutions, *Z*_*k*_ represents the genotype of animal *i* at SNP *k* and takes on the values of 1, 0, or -1, *g* contains the additive allele substitution effect of SNP *k*, *u*_*i*_ is the infinitesimal polygenic effect of animal *i* as estimated by Qxpak via a pedigree based additive animal model including three generations and a total of 933 individuals, and *e*_*ik*_ is the residual variance, with random effects distributed as multivariate normal with mean equal to 0.

In addition to the single SNP analyses, we also applied a model fitting the *NCAPG I442M* and *GDF8 Q204X* variants simultaneously (2 SNP model) and a further model fitting both variants and additionally testing for their interaction (2 SNP interaction model). Depending on the analyzed trait, different fixed effects were included in the above model: fixed effects for RFI and FCR were year of birth and season. The variable ‘season’ specified for each individual the season in which the animal spent most of the test period (season 1 = May to October, season 2 = November to April). For dEI and ADG only the year of birth was included as fixed effect, as season did not significantly affect the traits as determined in initial analyses. For the association studies of the metabolites, the year of sampling and the day of measurement were included as fixed effects.

#### Network approach

In contrast to monogenic traits, complex traits are affected by dozens or hundreds of genes with mostly very small effect sizes. Although GWAS are capable to detect loci with major effects on a complex trait, GWAS often lack the power to discriminate loci with small effect sizes from false positive associations. In our study, the two approaches association weight matrix (AWM, [32]) and partial correlation information theory (PCIT, [54]) were applied in order to combine the single trait GWAS results in a comprehensive network approach. AWM and PCIT were essentially set up as described in Widman et al. [12]. Initially, the traits were subdivided into a key trait and a number of supportive traits. The key trait is physiologically closest to the complex trait, and the information contributed from the supportive traits further enriches the analysis. In the present study, RFI was chosen as key trait, whereas FCR, dEI, and a subset of plasma metabolite concentrations were selected as supportive traits. Due to the strong phenotypic correlations within groups of metabolites [12] and in order to include a recommended maximum of independent information into the subsequent network approach, it became necessary to reduce the number of metabolites for the analysis. The final subset of metabolites (see Table 1) comprised the plasma concentration of the following components: arginine, lysine, C0, C2, C5, C8:1, C14, C18, PC_aa_C32:0, PC_ae_C36:1 and SMC_20:2. This selection was performed as described in Widmann et al. (2013), represents a set of lowly correlated metabolites and further focuses on metabolites with a strong impact of protein turnover (amino acids) or lipid metabolism (acylcarnitines, phosphocholines and sphingomyelins). As previously outlined, feed efficiency depends on a variety of biological parameters. Thus, GWAS results from FCR will contribute information about the animals’ body mass accretion properties, and GWAS data on dEI will add information about food consumption. After defining the key- and supportive traits, the subsequent statistical analyses were conducted essentially as described by Widmann et al. [12]. After normalizing the SNP effects from the GWAS for all traits, those SNPs were introduced into the AWM that showed an association with the key trait at a significance level p ≤ 0.05 (respective threshold as recommended by Reverter and Fortes [55]). Subsequently, the average number A_P_ of associations of those SNPs with the supportive traits also at p ≤ 0.05 was determined. Then, all SNPs that previously failed to be associated with the key trait, but that were associated to more than A_P_ supportive traits were additionally added into the AWM. All SNPs in the AWM were then filtered for those that were located closer than 2,500 bp to a gene or even within a gene. This step ensured that SNPs in the final AWM were either localized close or within functional regulatory or coding gene elements. For this purpose, all SNPs were mapped against the UMD3.1 assembly (ftp://ftp.cbcb.umd.edu/pub/data/assembly/Bos_taurus/Bos_taurus_UMD_3.1/, accessed: 24/01/2014). If a gene was represented by more than one SNP, then the only SNP that was kept was the one associated to the highest number of key- and supportive traits. The final AWM matrix was set up by assigning to each cell [*i*,*j*] of the matrix the additive SNP effect of the *i*th gene for the *j*th trait. Finally, AWM has identified a set of genes that is potentially relevant for the complex trait feed efficiency. The output of AWM is a matrix with rows containing genes with a potential impact on feed efficiency and with columns containing the examined key and supportive traits. The cells [*i*,*j*] of this matrix include information on size and direction of the additive gene/SNP effects for the key- and supportive traits. The AWM (see S1 Table) was visualized and analyzed with the software PermutMatrix version 1.9.3 [56].

**Table 1:** Descriptive statistics for the traits included in the analyses.

Trait	Acronym	Unit	N*	Average	Minimum	Maximum	Std Dev
Residual feed intake	RFI	MJ ME/kg body weight	175	0.02	-18.1	15.4	5.71
Feed conversion rate	FCR	MJ ME/kg weight gain	175	50.9	40.3	68.3	5.38
Daily energy intake	dEI	MJ ME/day	237	77.2	47.5	117	9.76
Average daily weight gain	ADG	kg/day	173	1.49	0.94	1.92	0.18
Arginine	Arg	μM	148	93.3	33.7	200	21.7
Free carnitine	C0	μM	149	6.12	3.49	9.99	0.85
Acetylcarnitine	C2	μM	147	0.96	0.39	2.73	0.36
Valerylcarnitine	C5	μM	147	0.064	0.026	0.130	0.018
Suberylcarnitine	C81	μM	147	0.006	0.001	0.139	0.011
Myristylcarnitine	C14	μM	147	0.011	0.003	0.027	0.004
Stearoylcarnitine	C18	μM	147	0.021	0.007	0.069	0.011
Diacylphosphatidylcholine C32:0	PC_aa_C32:0	μM	146	4.72	1.28	9.30	1.65
Acylethylphosphatidylcholine C36:1	PC_ae_C36:1	μM	146	13.4	4.5	31.6	5.1
Sphingomyelin C20:2	SM_C20:2	μM	146	3.44	0.70	7.71	1.52

*Number of animals included in the analysis

The AWM was subsequently processed with the PCIT algorithm [54] to determine significant correlations among the AWM genes, based on their additive SNP effect sizes, which in turn will define edges in the reconstruction of the co-association network. Briefly, PCIT identifies relevant gene-gene associations between all genes in the AWM by comparing the first-order partial correlations (PCs) of gene pairs with the PC of any other gene in the matrix. By applying an information theory approach, PCIT further determines thresholds for significant gene-gene interactions based on the established PCs. Especially this last step makes PCIT appealing for the analysis of co-association networks, because thresholds for significance are ascertained from the data itself, while simultaneously avoiding a subjectively chosen threshold. Finally, by connecting the significantly correlating genes, the determined gene-gene relationships were visualized in a network structure, where every node represents a putatively relevant gene for feed efficiency and every connection between two genes represents significantly correlated SNP effects. In order to further confirm that the resultant network contained biologically meaningful information instead of being just a random accumulation of genes, 10 completely random networks were created and compared with the original network. For this purpose, the standardized additive effects in the AWM matrix were independently randomized ten times, resulting in ten random AWM matrices with SNP-trait associations completely independent from the associations obtained by the original GWAS results.

#### Network analysis

As indicated in the previous section, the network established by AWM and PCIT contained genes that might modulate feed efficiency in the SEGFAM resource population and interactions between those genes based on additive gene/SNP effects. The network was visualized with the software Cytoscape version 3.1.0 [57]. Given that PCIT creates a very complex dataset of gene-gene interactions, the present study only analyzed significant interactions with a |PC| ≥ 0.80, according to PCIT. This subset of data represents an acceptable balance between the number of significant interactions and the amount of data that could efficiently be analyzed with Cytoscape. For the topological analysis of the feed efficiency network, we used the Network Analyzer plugin [58]. The functional annotation tool in DAVID 6.7 [59], implementing a hypergeometric test and correcting for multiple testing according to Benjamini and Hochberg [60], was applied for the identification of overrepresented Gene Ontology (GO) terms. The required reference list for testing the enrichment of genes from the AWM/PCIT network comprised all genes from the UMD3.1 assembly that were represented via a SNP from the Illumina Bovine SNP50 v2 within 2,500 bp. The distance of 2,500 bp was selected as a very conservative threshold, because the respective chromosomal region can be assumed to contain the promoter region of a gene, and the threshold also enabled an almost unambiguous assignment of a SNP to a single gene. The human genome served as reference annotation due to its superior quality compared to the bovine annotation. To confirm and expand a DAVID result, functional network analyzes were additionally performed with the Ingenuity pathway analysis software (IPA, Ingenuity Systems, http://www.ingenuity.com/). In contrast to DAVID, functional analyses conducted in IPA exploit the Ingenuity Pathways Knowledge Base which is a manually curated database containing canonical pathways and gene-gene interaction data obtained from previously published data.

## Results

### *NCAPG* I442M and *GDF8* Q204X association analyses

The present study investigated the impact of the two loci *NCAPG I442M* (*rs109570900*) and *GDF8 Q204X* (*rs110344317*) on feed efficiency and its related traits in male cattle. The effects of *NCAPG I442M* and *GDF8* Q204X were tested for associations with RFI, FCR, dEI, ADG and plasma levels of arginine and free carnitine. Descriptive statistics for the phenotypic data are given in Table 1. Due to its distributional property RFI is expected to have an average of zero [RFI ~ N(0, σ^2^_RFI_)] [14]. Our association analyses revealed that the *NCAPG I442M* locus was significantly associated with RFI, FCR, dEI, ADG and plasma levels of arginine (Table 2). The strongest associations were observed between *NCAPG I442M* and ADG (p = 4.54 x 10^–6^) and RFI (p = 1.27 x 10^–5^). Our analyses further show that the *442M* allele significantly decreases RFI and FCR, whereas it significantly increases the animals’ energy intake, their weight gain and their plasma levels of arginine (Table 2). Regarding *GDF8 Q204X*, the analyses show that *GDF8 Q204X* is significantly associated with RFI (p = 4.55 x 10^–4^) and plasma levels of free carnitine (p = 3.02 x 10^–4^, Table 2). The growth-promoting *GDF8 204X* allele decreases the animals’ RFI, FCR and plasma carnitine levels. Fitting both loci (*NCAPG I442M* and *GDF8 Q204X*) simultaneously into a model (2 SNP model) did not change the associated effects compared to the single-SNP model (Table 2) indicating that the two loci act independently. This was further confirmed by the lack of an indication on any significant interaction effect between the two loci in the 2 SNP interaction model for RFI, FCR, dEI, ADG and plasma levels of arginine or C0 (Table 2).

**Table 2:** Results of the *NCAPG* I442M and *GDF8* Q204X association analyses.

	Single marker analysis				2 SNP model			2 SNP interaction model
	NCAPG I442M^1^		GDF8 Q204X^2^		NCAPG I442M^1^	GDF8 Q204X^2^		vs. 2 SNP model	
	a^3^	p-value	a^3^	p-value	a^3^	a^3^	p-value	p-value^4^	
RFI	-2.18 (0.49)	0.13 x 10^–4^	-3.30 (0.91)	0.46 x 10^–03^	-2.04 (0.47)	-3.07 (0.87)	0.37 x 10^–06^	0.92	
FCR	-1.85 (0.50)	0.12 x 10^–03^	-1.99 (0.96)	0.04	-1.76 (0.50)	-1.79 (0.92)	0.14 x 10^–03^	0.44	
dEI	1.62 (0.70)	0.03	-1.09 (1.31)	0.38	1.39 (0.74)	-1.11 (1.31)	0.14	0.36	
ADG	0.08 (0.02)	0.45 x 10^–05^	0.06 (0.03)	0.06	0.08 (0.02)	0.06 (0.03)	0.81 x 10^–05^	0.13	
Arg	9.03 (2.35)	0.19 x 10^–03^	-6.20 (4.68)	0.19	9.54 (2.34)	-8.78 (4.46)	0.14 x 10^–03^	0.29	
C0	0.11 (0.09)	0.26	-0.73 (0.17)	0.25 x 10^–04^	0.14 (0.09)	-0.77 (0.17)	0.42 x 10^–04^	0.08	

^1^ rs109570900

^2^ rs110344317

^3^ allele substitution effect (standard error) NCAPG 442M vs. 442I or GDF8 204X vs 204Q, respectively

^4^ p-value testing a model fitting both SNPs and their epistatic interaction vs. a model fitting both SNPs

### GWAS

In the present study, 176 male SEGFAM bulls were genotyped at 54,609 genetic loci. After filtering for call rates and minor allele frequencies, the SNP dataset for the subsequent GWAS and network analyses comprised 44,507 high quality SNPs. Single-trait-single-SNP GWAS were run for RFI, FCR, dEI and 221 metabolites. Manhattan plots of the GWAS results for RFI, FCR, dEI and the subset of metabolites selected as supportive traits are given in S1 Fig. The GWAS for RFI confirms the observed association between *NCAPG* and RFI (Table 2). *NCAPG I442M*, located in the middle region of BTA6, is the most strongly associated SNP for RFI, followed by the SNP BTB-02002785 also on BTA6 located upstream from *NCAPG I442M* (S1A Fig). We further confirmed previously reported associations [8] between the middle region of BTA6 and the plasma level of arginine (S1 Fig) and between the centromeric region of BTA2 and the plasma levels of free carnitine (S1 Fig). The strongest overall association was observed between the SNP BTB-00081518, located in the centromeric region on BTA2, and the metabolite SMC_20:2 (p = 2.97 x 10^–7^; S1 Fig].

### Network analyses

**Fig 1:**
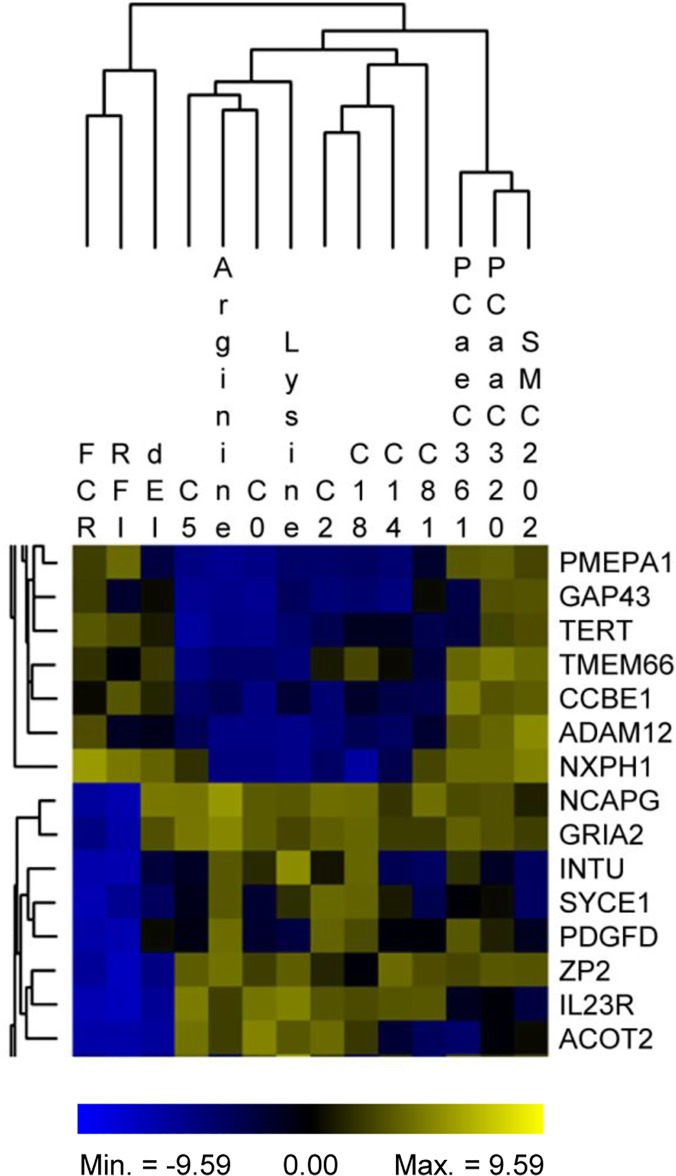
Subset of the association weight matrix (AWM). Column wise, the AWM compares correlations between phenotypes, and row wise AWM compares gene-gene interactions. Cells within the matrix correspond to normalized additive effects of gene-associated SNPs as obtained from genome-wide association studies. Squares of blue and yellow color gradients visualize the strength of standardized additive gene (SNP) effects. Abbreviations of phenotypic traits as defined in Table 1.

After running single trait GWAS for the 14 selected traits (see Table 1), the results were simultaneously integrated and evaluated in a network approach. The final aim of this approach was the detection of genes and gene-gene interactions that are likely participating in the modulation of feed efficiency at the onset of puberty in male SEGFAM cattle. By applying the AWM approach we detected a total of 985 genes potentially relevant for feed efficiency (S1 Table). When focusing on the column wise tree cluster of the AWM, specifying the 14 analyzed traits, it becomes apparent that the traits grouped into four subclusters (Fig 1). The first subcluster comprised phenotypic traits directly related to feed intake and efficiency (RFI, FCR, dEI), the second subcluster comprised the plasma amino acid levels for arginine and lysine and for two short chain acylcarnitines (C0, C5), the third subcluster comprised the plasma levels for medium and long chain acylcarnitines (C8:1, C14, C18) and for C2, and the fourth subcluster comprised plasma levels for phosphocholines (PC_aa_C32:0, PC_ae_C36:1) and a sphingomyelin.

**Fig 2:**
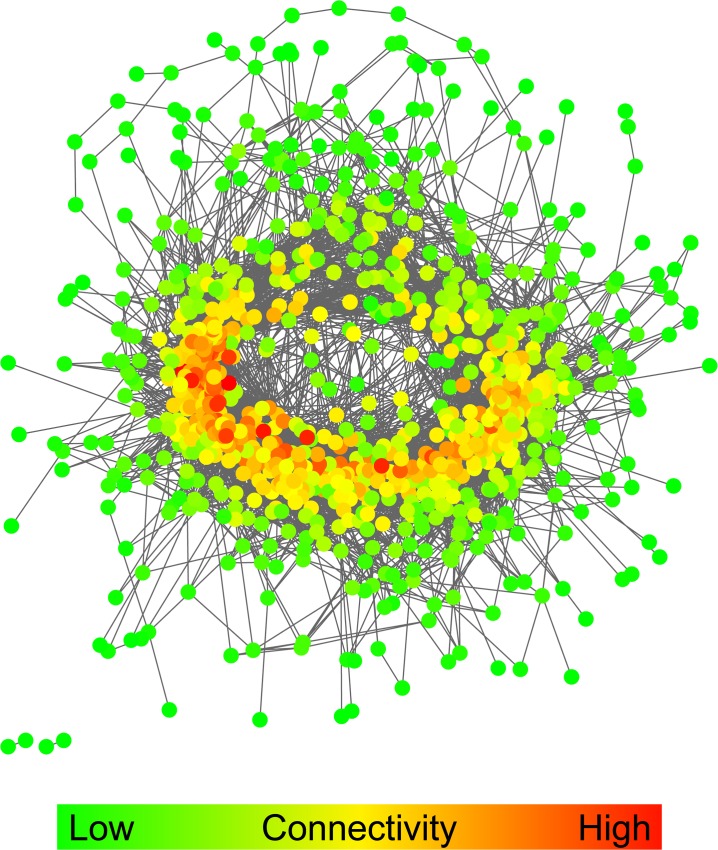
The feed efficiency network. Each dot in the network represents a gene putatively relevant for variation in feed efficiency. Genes are colored according to their connectivity in the network ranging from 1 connection (dark green) to 99 connections (dark red). Edges (grey lines) connect genes which display significantly correlating additive effects for the quantitative trait. Genes and edges were determined by AWM and PCIT procedures, respectively. In sum, the network comprises 955 genes which are connected by 10,927 edges.

**Fig 3:**
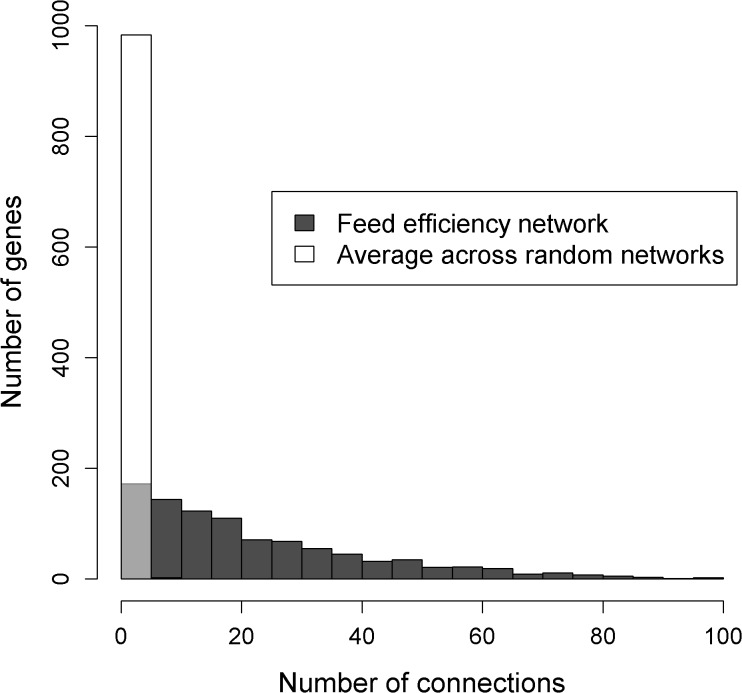
Topological comparison between the feed efficiency network and 10 randomly generated networks. The figure illustrates the number of connections per gene in the feed efficiency network and the average number of connections per gene across the ten random networks. Due to the transparent style of the white bars, black bars or parts of black bars that are hidden by a white bar are colored in light grey.

Based on the AWM result, the subsequent PCIT analysis determined the significant interactions between genes in the AWM based on the strength of their partial correlation. In total, PCIT identified 72,023 significant correlations among the 985 genes included in the AWM implying a clustering coefficient of 14.9% of all the 484,620 possible connections among the 985 genes. Because this large amount of data was difficult to handle with Cytoscape and its implemented add-ons, we kept only the most significant interactions (i.e., all interactions with |PC| > 0.80) for the final functional analyses. However, this subset still comprised 955 genes displaying 10,927 direct interactions (S2 Table). The resulting feed efficiency network is visualized in Fig 2. Compared to the number of genes in the feed efficiency network, all of the ten random networks were considerably smaller with only 552 genes and 1,050 interactions on average. The feed efficiency network and the random networks clearly differed in their topologies as the feed efficiency network was much denser interconnected (Fig 3). Within the feed efficiency network, the connectivity degree per gene (i.e. the number of further genes to which a single gene is connected) was continuously distributed and ranged from 1 to 99 connections (Fig 3). In contrast to this, connections in the random networks were much less variable with nearly all genes possessing five or less connections on average (Fig 3). In sum, 316 genes in the feed efficiency network were connected to ≤ 10 further genes, and 10 genes were connected to ≥ 80 further genes. The latter, so called “hubs” in the feed efficiency network, comprised the genes *LIMK2*, *SRGAP1*, *ANKRD40*, *UGCGL2*, *PARK2*, *FYN*, *FAM48A*, *RIMS3*, *AGPS* and *SESTD1* (Table 3).

**Table 3:** The 10 most densely connected genes in the feed efficiency network.

ID	Official gene name	Chromosome	Position [UMD 3.1]	Connectivity
*LIMK2*	LIM domain kinase 2	17	72,142,468–72,196,938	99
*SRGAP1*	SLIT-ROBO Rho GTPase activating protein 1	5	49,807,611–50,119,313	96
*ANKRD40*	Ankyrin repeat domain 40	19	36,655,574–36,675,087	93
*UGCGL2*	UDP-glucose glycoprotein glucosyltransferase 2	12	77,032,463–77,176,980	90
*PARK2*	Parkin RBR E3 ubiquitin protein ligase	9	98,421,510–98,453,799	89
*FYN*	FYN oncogene related to SRC, FGR, YES	9	39,047,795–39,260,856	88
*FAM48A*	FAM48A family with sequence similarity 48, member A	12	24,668,828–24,708,465	85
*RIMS3*	Regulating synaptic membrane exocytosis 3	3	106,132,498–106,175,009	83
*AGPS*	Alkylglycerone phosphate synthase	2	19,339,167–19,433,176	82
*SESTD1*	SEC14 and spectrin domains 1	2	17,626,648–17,731,390	81

**Fig 4:**
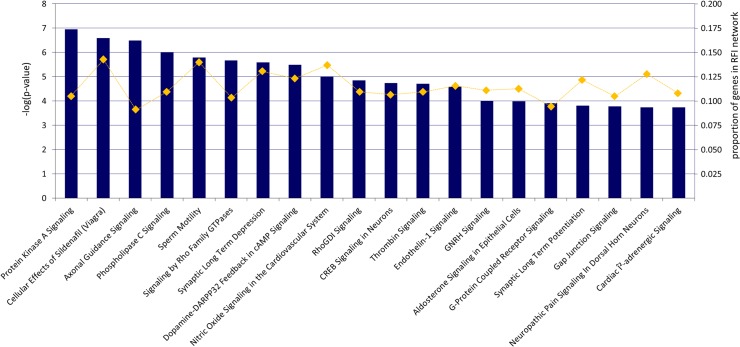
Overview over the most highly enriched canonical pathways in the feed efficiency network. Bars represent canonical pathways as labeled. The lengths of the bars reflect the strength of association [-log(pvalue)]. In total, the feed efficiency network was significantly enriched for 102 canonical pathways [-log(pvalue) ≥ 1.3]. A summary of all significantly enriched canonical pathways is given in S5 Table. Canonical pathways were determined with the Ingenuity Pathway Analysis tool (IPA). As input for the analysis served all 936 genes from the feed efficiency network that could successfully be annotated by IPA. Correction for multiple testing was carried out via a Fisher’s exact test as implemented in IPA.

**Fig 5:**
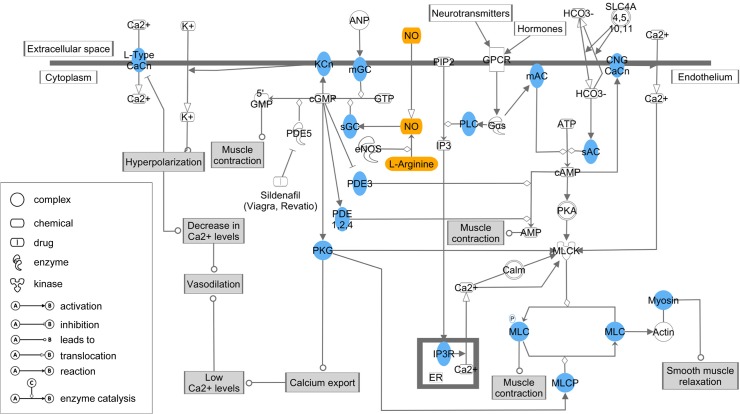
Schematic view on the canonical pathways “Cellular effects of Sildenafil”. Components highlighted in blue are coded by genes from the feed efficiency network. L-arginine and nitric oxide are colored in orange. Physiological reactions are colored in grey. Graph adapted from IPA.

The feed efficiency network was further scrutinized with the functional annotation tools DAVID and IPA. These analyses aimed at the detection of common pathways and modulators that might affect variation in feed efficiency due to their dependency on genes from the feed efficiency network. Our functional analyses with DAVID revealed that a single GO function, namely “calcium ion binding”, was significantly enriched with network genes (p = 4.53 x 10^–6^, S3 Table). In detail, a subset of 105 network genes, representing approximately 11% of all network genes, act in this function. Among these 105 loci were genes that code for, e.g., cadherins, calcium channel subunits, phospholipases and protein kinases. The analyses with IPA additionally revealed that 100 canonical pathways were significantly enriched with genes originating from the feed efficiency network (S4 Table). The overall highest enriched pathways were the “Protein kinase A signaling” and the “Cellular effects of Sildenafil” pathways (Fig 4). In the “Cellular effects of Sildenafil” pathway, a total of 16 components acting in this pathway were coded by genes from the feed efficiency network (Fig 5). Fig 5 further illustrates how these components interact with each other and with the two metabolites arginine and nitric oxide (NO) in order to promote the physiological reaction of smooth muscle relaxation. Regarding biological functions, IPA analysis revealed several categories related to cellular function and maintenance (including cell morphology, organismal survival, cellular assembly and organization and cellular function and maintenance) that were enriched with genes for the RFI network (Table 4). Finally, IPA determined a set of transcriptional regulators (upstream regulators) that potentially affect the expression of network genes and thus might have an impact on feed efficiency. In total, IPA identified 314 upstream regulators with significant regulative effects on feed efficiency related genes (p-value ≤ 0.05, S5 Table). In this list, the two regulators *TP53 (Tumor protein p53)* and *TGFB1* (*transforming growth factor*, *beta 1*) are the most significant regulators associated with feed efficiency genes (p-value of overlap with network genes = 2.08 x 10^–9^ and 5.6 x 10^8^, respectively). In total, *TGFB3* and *TGFB1* overlapped with 170 network genes (approximately 17.8% of all genes in the RFI network), illustrating the major importance of these two transcription or growth factors for the regulation of feed efficiency.

**Table 4:** Top biological categories enriched with genes from the RFI network as determined by IPA.

Category	p-value
Cell Morphology	2.03 x 10^–16^ to 4.93 x 10^–03^
Organismal Survival	2.07 x 10^–15^ to 1.04 x 10^–06^
Cellular Assembly and Organization	6.53 x 10^–14^ to 4.4 x 10^–03^
Cellular Function and Maintenance	6.53 x 10^–14^ to 4.17 x 10^–03^
Nervous System Development and Function	4.44 x 10^–13^ to 4.65 x 10^–03^
Tissue Morphology	2.65 x 10^–12^ to 4.44 x 10^–03^
Behavior	4.84 x 10^–12^ to 4.7 x 10^–03^
Cell-To-Cell Signaling and Interaction	7.98 x 10^–12^ to 4.4 x 10^–03^
Organismal Development	5.4 x 10^–11^ to 4.65 x 10^–03^
Embryonic Development	4.29 x 10^–10^ to 4.34 x 10^–03^
Organ Development	4.29 x 10^–10^ to 4.34 x 10^–03^
Tissue Development	4.29 x 10^–10^ to 4.92 x 10^–03^
Cardiovascular System Development and Function	1.23 x 10^–08^ to 4.17 x 10^–03^
Cardiovascular Disease	1.26 x 10^–08^ to 3.96 x 10^–03^
Cellular Development	1.3 x 10^–08^ to 4.93 x 10^–03^
Cellular Movement	1.22 x 10^–07^ to 4.4 x 10^–03^
Cancer	1.22 x 10^–07^ to 4.66 x 10^–03^
Organ Morphology	3.26 x 10^–07^ to 4.77 x 10^–03^
Hereditary Disorder	6.82 x 10^–07^ to 1.93 x 10^–03^

RFI: residual feed intake

## Discussion

Our data revealed independent, significant effects of both the *NCAPG I442M* (*rs109570900*) and the *GDF8 Q204X* (*rs110344317*) loci on residual feed intake and feed conversion. For both loci, the growth promoting allele at the onset of puberty was associated with a negative effect on residual feed intake. Thus, individuals carrying the *NCAPG 442M* or the *GDF8 204X* allele had an increased feed efficiency as indicated by the direction of the allele substitution effects for *NCAPG I442M* and *GDF8 Q204X*. The increased feed efficiency was also reflected by a better feed conversion ratio associated with both alleles. Whereas for the *GDF8 Q204X* locus, no significant effect on daily energy intake was observed, at the *NCAPG I442M* locus the *442M* allele was also associated with an elevated energy intake. An increased dry matter intake associated with the *NCAPG 442M* allele had already been described by Lindholm-Perry et al. [13] in a crossbred beef cattle population. The increased energy intake of the *NCAPG 442M* allele promoting growth and improving feed efficiency may be interpreted as a dominating effect of the locus on growth. The elevated energy demand for increased growth triggered by the *NCAPG 442M* allele is not fully compensated for by an increased efficiency in converting feed into body tissue. As a consequence, the individuals carrying the *NCAPG 442M* allele had an additional demand for energy uptake that is reflected by the association of the allele with increased daily energy intake as observed in our study. The divergent effect of the *NCAPG I442M* locus on residual feed intake and average daily energy intake is in contrast to the overall positive phenotypic correlations between RFI and dEI in this study and also in the literature [16], which indicates that feed efficiency was unfavorably correlated with daily energy intake. Thus, the *NCAPG I442M* locus-specific, favorable correlation between both traits is in contrast to the unfavorable phenotypic correlations reflecting the action of the entire genome (Table 5). This contrasting feature makes this SNP an excellent candidate for marker-assisted selection programs. A similar observation about a contrast between locus-specific and global correlations for phenotypic traits had also been obtained at the *diacylglycerol O-acyltransferase 1 (DGAT1) K232A* locus for milk fat and milk protein yield in dairy cattle [19,21].

**Table 5:** Correlations of phenotypic data (above diagonal) vs. correlations of 44,506 SNP allele substitution effects (below diagonal).

	RFI	FCR	dEI	Arg	Lys	C0	C2	C5	C8:1	C14	C18	PC_aa_C32:0	PC_ae_C36:1	SMC_20:2
RFI		.67	.60	-.05	.17	-.03	-.11	-.06	.04	-.10	-.11	.13	.18	.23
FCR	.63		.36	-.27	-.05	-.09	-.04	-.14	.00	.12	-.05	.01	.01	.10
dEI	.36	.42		.08	.32	-.23	-.22	-.03	.11	-.16	-.08	.08	.13	.20
Arg	.02	.01	-.01		.35	.43	.26	.48	.08	.02	.04	.10	.14	.03
Lys	.02	.02	.01	.33		.17	-.14	.29	-.11	-.10	-.02	-.03	.05	.08
C0	.01	.00	.01	.43	.31		.59	.40	-.07	.28	.26	.06	-.01	-.02
C2	.01	.01	.00	.23	.07	.55		.10	.00	.33	.52	.22	.15	-.03
C5	.00	.01	.00	.48	.19	.44	.07		.02	.04	-.12	-.12	.02	-.11
C8:1	.00	.01	-.01	.00	-.11	.08	.13	.05		.03	.09	.06	.06	.00
C14	.00	.02	.00	.02	.04	.23	.36	.07	.12		.56	.03	.04	.01
C18	.01	.02	.00	.03	.10	.22	.64	-.16	.14	.53		.01	.07	-.08
PC_aa_C32:0	.00	.00	.00	.07	-.02	.05	.23	-.10	.12	.15	.11		.84	.86
PC_ae_C36:1	.00	.00	.00	.13	.00	.03	.20	.03	.14	.12	.12	.87		.75
SMC_20:2	.00	.00	.01	.03	.02	.03	.13	-.06	.07	.15	.05	.89	.77	

for trait abbreviations see Table 1

In contrast to the study on the specific potential effects of the *NCAPG I442M* and *GDF8 Q204X* loci, network analyses do not focus on the effect of a specific mutation but thrive to highlight genes, networks and pathways relevant to the target trait. This information can subsequently be incorporated into focused refined association analyses [61]. When comparing the full RFI network from this study to the previously published growth-related network obtained for the same ontogenetic stage (onset of puberty, [12], there is a fundamental difference in the central hubs of the two networks. None of the ten most densely connected genes in the RFI network (Table 3) is in the respective list of hubs for the growth-related network, although the same metabolites were included as supportive traits. However, when looking at the significantly enriched canonical pathways containing genes from the RFI network, there is some overlap with the growth network. The GnRH signaling pathway, which was the most significantly enriched KEGG pathway for the growth network, is also in the list of the most significantly affected canonical pathways in the RFI network. The most distinct mechanism, however, for the RFI network highlighted by an affected canonical pathway indicated from the Ingenuity Pathway Analysis is the NO signaling (“cellular effects of Sildenafil”, “NO signaling in the cardiovascular system”). In contrast, although a regulatory role of NO had been postulated for the *NCAPG* growth-related sub-network, this was not the case for the total growth network in our previous study [12]. Together with the unique hubs for the RFI network, this demonstrates the specificity of the RFI network and underlines previous studies reporting that at the whole genome level growth and RFI are genetically independent [16,18]. In contrast, for the *NCAPG* locus, a connection between genetic variance affecting growth and feed efficiency-related processes is demonstrated by the significantly associated effects for both traits in the corresponding GWAS.

The hubs of the RFI network, which had been obtained solely from the data generated in this study, contain several genes with a role in cell communication (e.g., *SLIT-ROBO Rho GTPase Activating Protein 1 (SRGAP1)*, involved in the SLIT-ROBO signaling pathway). This fits the independent observation provided by IPA that the RFI network is significantly enriched with genes from the biological functions “Cell morphology” and “Cellular assembly and organization” (Table 4). By definition, RFI is the difference between the actual feed intake of an individual and its expected feed requirements for body maintenance and growth [14]. Our data about biological processes enriched with genes from the RFI network suggest that genetic variation in RFI is driven by genetic modulation of basic processes relevant to general cellular functions. The most strongly biological functions enriched with genes from the RFI network affected are Cell Morphology, Organismal Survival and Cellular Assembly and Organization. This is in line with most strongly enriched canonical pathway (Protein kinase A signaling), which plays a key role in all of these processes. Interestingly, there is a positional coincidence of two major hubs (*Alkylglycerone phosphate synthase*, *AGPS; SEC14 and spectrin domains 1*, *SESTD1)* in the RFI network and a major locus affecting sphingomyelin C20:2 (S1 Fig) in the chromosomal region of BTA2 at 17–20 Mb. *AGPS* and *SESTD1* are both known for their relevance in phospholipid synthesis. Besides their importance for cell wall structure, there is a growing evidence for a substantial role of sphingomyelins in energy metabolism [62] suggesting that modulation of sphingolipids via the central hubs *AGPS* and *SESTD1* in the RFI network might be relevant for genetic variation in feed efficiency.

When looking at the predicted upstream regulators from the RFI network, the *TP53* and *TGFB1* genes stood out regarding significance of overlap and number of target molecules in the dataset (Table 6). There are several reports suggesting that *TP53* might be a relevant factor modulating feed efficiency. The *TP53* gene was reported as a member of a phenotype-driven GWAS-based feed efficiency network in cattle by Serão et al. [28]. The high relevance of *TP53* as upstream regulator involved in feed efficiency was also supported by Chen and co-workers [31]. The group found the central transcription factor *TP53* in a gene network obtained from global transcriptome analysis between high and low RFI cattle. Analogous data were obtained in a porcine model combining responses to caloric restriction and RFI in liver and fat gene expression analyses [63]. There, *TP53* was among the transcription factors with the greatest number of connections to differentially expressed genes. These results further support the hypothesis suggested by our combined phenotype-metabolome-genome systems biology analysis that *TP53* is a major upstream regulator for genetic variation of RFI. Another significant upstream regulator identified in our dataset for the RFI network is *SETDB1*, which has been found to be associated with anabolic function and was among the most significantly upregulated genes in a high feed efficiency chicken broiler line [64].

**Table 6:** Upstream regulators in the RFI network obtained by Ingenuity Pathway Analysis.

Upstream Regulator	Molecule type^a^	p-value of overlap^b^	Target molecules in RFI network^c^
*TP53*	transcription regulator	2.08 x 10^–09^	95
*TGFB1*	growth factor	5.60 x 10^–08^	106
*ERG*	transcription regulator	1.66 x 10^–06^	22
*Sos*	group	4.41 x 10^–06^	23
*PKD1*	ion channel	1.22 x 10^–05^	20
*TGFB3*	growth factor	1.44 x 10^–05^	14
*HNRNPA2B1*	other	3.65 x 10^–05^	17
*PADI2*	enzyme	5.91 x 10^–05^	3
*RB1*	transcription regulator	6.65 x 10^–05^	24
*ZNF217*	transcription regulator	1.02 x 10^–04^	11
*TBX5*	transcription regulator	1.15 x 10^–04^	10
*ERBB2*	kinase	2.01 x 10^–04^	43
*ER*	group	2.35 x 10^–04^	21
*REST*	transcription regulator	2.71 x 10^–04^	13
*NFE2L2*	transcription regulator	3.14 x 10^–04^	30
*DMD*	other	4.19 x 10^–04^	17
*DAB1*	other	5.57 x 10^–04^	3
*SETDB1*	enzyme	7.60 x 10^–04^	6
*FOS*	transcription regulator	9.81 x 10^–04^	34
*WNT3A*	cytokine	1.04 x 10^–03^	18

^a^ the molecule class to which the respective upstream regulator belongs

^b^ p-value of significance as determined from the number of feed efficiency genes that overlap with the respective upstream regulator

^c^ number of RFI network genes targeted by the respective upstream regulator

RFI: residual feed intake.

*TGFB1* and *TGFB3*, also listed among the top upstream regulators, both belong to the transforming growth factor family that comprises proteins with an important role in control of proliferation and differentiation. Several *TGFB1* knockout mice models show a wasting syndrome indicating severely disturbed energy metabolism (e.g., Shull et al. [65] demonstrating the functional impact of *TGFB1* on feed utilization. In addition, *TGFB1* is a paralogue gene to the well-established growth-modulating transcription factor *GDF8*.

No significant correlation, neither phenotypically nor for SNP-associated effects were observed between arginine and RFI (Table 5) indicating that the genetic effects on RFI were not modulated via arginine. This is in contrast to the significant and positive correlations between plasma arginine and growth at the onset of puberty (Table 5) and again demonstrates that both traits (RFI and daily weight gain) at the onset of puberty are modulated via divergent physiological mechanisms.

In conclusion, our data indicate a substantial favorably correlated effect of the *NCAPG I442M* locus on feed efficiency and feed intake in male cattle at the onset of puberty. Whether this effect will also be present in females, e.g., during lactation will have to be determined. However, due to QTL for growth [1] and milk production [66] detected within or in the vicinity to the NCAPG gene, this chromosomal region might be regarded as potential switch for nutrient partitioning in cattle. For the RFI network, our results confirm the *TP53* and *TGFB1* genes as key modulators of feed efficiency. Furthermore, our RFI network provides further evidence for recently built hypotheses [62] suggesting a functional relevance of sphingolipids to energy metabolism. This proves the merit of exploiting metabolites as intermediate phenotypes that represent genetically determined links between the genome and an animals’ physiological status or a complex trait. Due to the strategy of generating the RFI network fully independent from information in other species, our data contribute a truly novel piece of evidence for the hypothesis of a functional relevance of sphingolipids for energy metabolism in growing cattle.

## Supporting Information

S1 Fig Manhattan plots resulting from the GWAS for the key trait and 13 supportive traits. Manhattan plots resulting from the GWAS for (A) residual feed intake (RFI), (B) feed conversion rate (FCR), (C) daily energy intake (dEI), (D) arginine, (E) lysine, (F) free carnitine (C0), (G) acetylcarnitine (C2), (H) valerylcarnitine (C5), (I) suberylcarnitine (C8:1), (J) myristylcarnitine (C14), (K) stearoylcarnitine (C18); (L) diacylphosphatidylcholine C32:0 (PC_aa_C32:0), (M) acylethylphoshatidylcholine C36:1 (PC_ae_C36:1), (N) sphingomyelin C20:2 (SM_C20:2). Manhattan plots were plotted by the R package qqman [67].
(PDF)

S1 Table The final AWM containing genes associated with feed efficiency. Matrix cells contain standardized additive gene effects. The file can be uploaded in PermutMatrix for visualization.
(TXT)

S2 Table Cytoscape input file to create the feed efficiency network. Genes in the file were determined by the combined approaches of AWM and PCIT. The first two columns contain the names of the genes that were tested for an interaction due to the partial correlation of their standardized additive gene effects. The third column contains the partial correlation as determined by PCIT. For visualization, the file can be uploaded in Cytoscape.
(TXT)

S3 Table Results from the functional analysis with DAVID. The table includes all molecular functions that the feed efficiency network was enriched for at a nominal significance level of p ≤ 0.05.
(TXT)

S4 Table Canonical pathways significantly enriched with genes from the RFI network. Summary of significantly enriched canonical pathways in the feed efficiency network [-log(pvalue) ≥ 1.3]. Canonical pathways were determined with the Pathway Analysis tool in IPA. Significance values were corrected for multiple testing via a Fisher’s Exact test. Ratio specifies the number of genes in a given pathway that meet the cutoff criteria, divided by total number of genes that make up the respective pathway.
(TXT)

S5 Table Significant upstream regulators in the RFI network. Result of the upstream regulator analysis in IPA. The first column of the file contains all upstream regulators that affect a significant number of feed efficiency genes (p ≤ 0.05), the second column specifies the molecule class to which the respective upstream regulator belongs, the third column contains the respective p-value of significance as determined from the number of feed efficiency genes that overlap with the respective upstream regulator and the fourth column contains the network genes targeted by the respective upstream regulator. Correction for multiple testing was conducted via a Fishers Exact test.
(TXT)
