# Supplementary material for: Systems Biology Analysis Merging Phenotype, Metabolomic and Genomic Data Identifies Non-SMC Condensin I Complex, Subunit G (NCAPG) and Cellular Maintenance Processes as Major Contributors to Genetic Variability in Bovine Feed Efficiency
Source: PLoS One. 2015 Apr 15;10(4):e0124574. doi: 10.1371/journal.pone.0124574 (PMC4398489; doi:10.1371/journal.pone.0124574)

Additional_file_1: Manhattan plots of GWAS results. Shown is the significance of association [-log10(p)] between SNPs across the bovine genome and the target traits from the two groups feed efficiency (A-C) and metabolites (D-N). The red line marks the significance level of p=0.05 before correction for multiple testing. SNPs above this line theoretically had the chance to be represented via a gene in the AWM.

(A) residual feed intake (RFI), **(B)** feed conversion rate (FCR), **(C)** daily energy intake (dEI), **(D)** arginine, **(E)** lysine, **(F)** free carnitine (C0), **(G)** acetylcarnitine (C2), **(H)** valerylcarnitine (C5), **(I)** suberylcarnitine (C8:1), **(J)** myristylcarnitine (C14), **(K)** stearoylcarnitine (C18); **(L)** diacylphosphatidylcholine C32:0 (PC_aa_C32:0), **(M)** acylethylphoshatidylcholine C36:1 (PC_ae_C36:1), **(N)** sphingomyelin C20:2 (SM_C20:2).

(A) Residual feed intake (RFI)

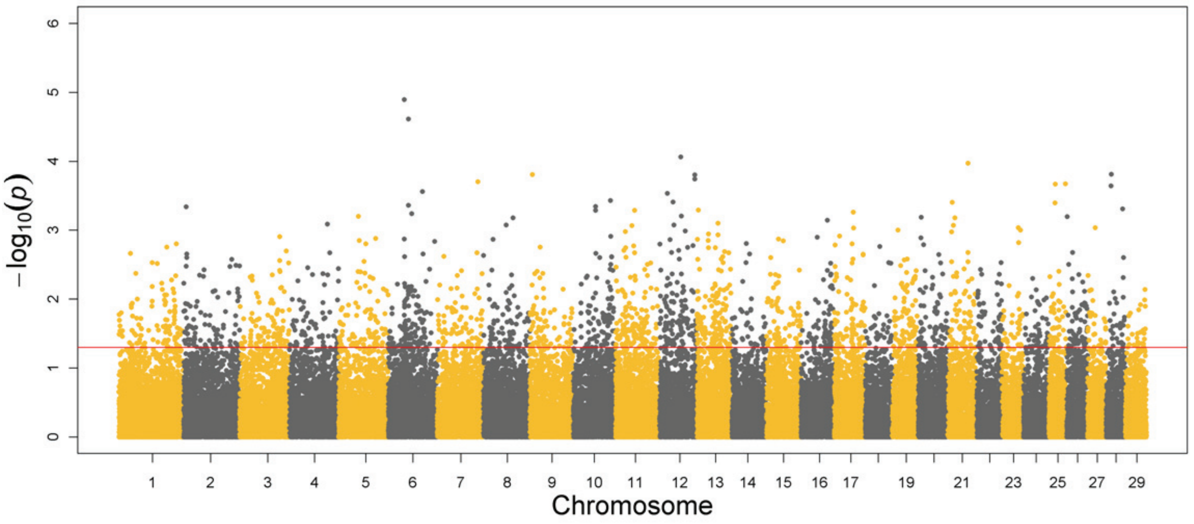

(B) Feed conversion rate (FCR)

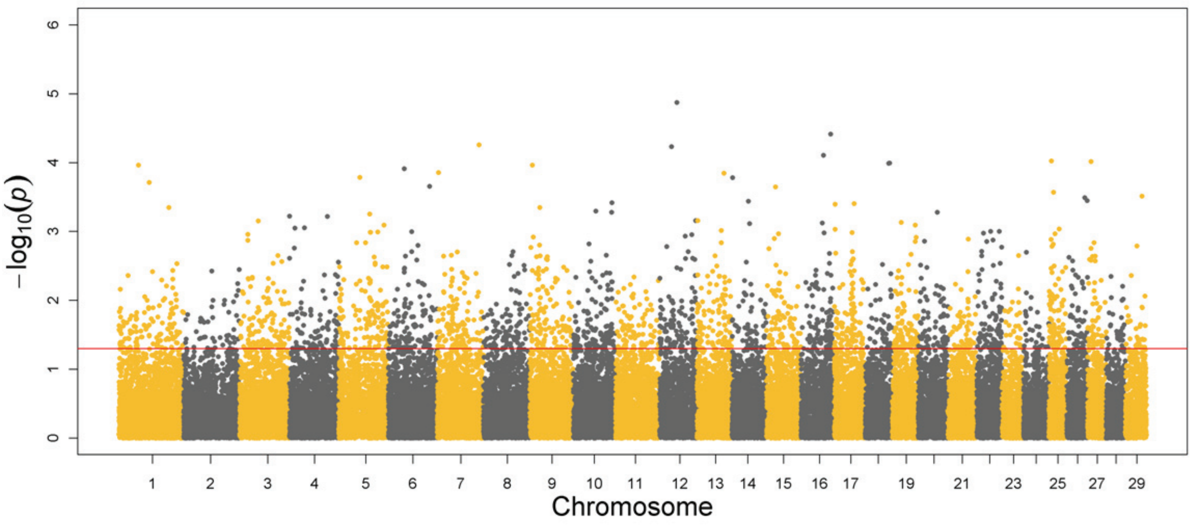

(C) Daily energy intake (dEI)

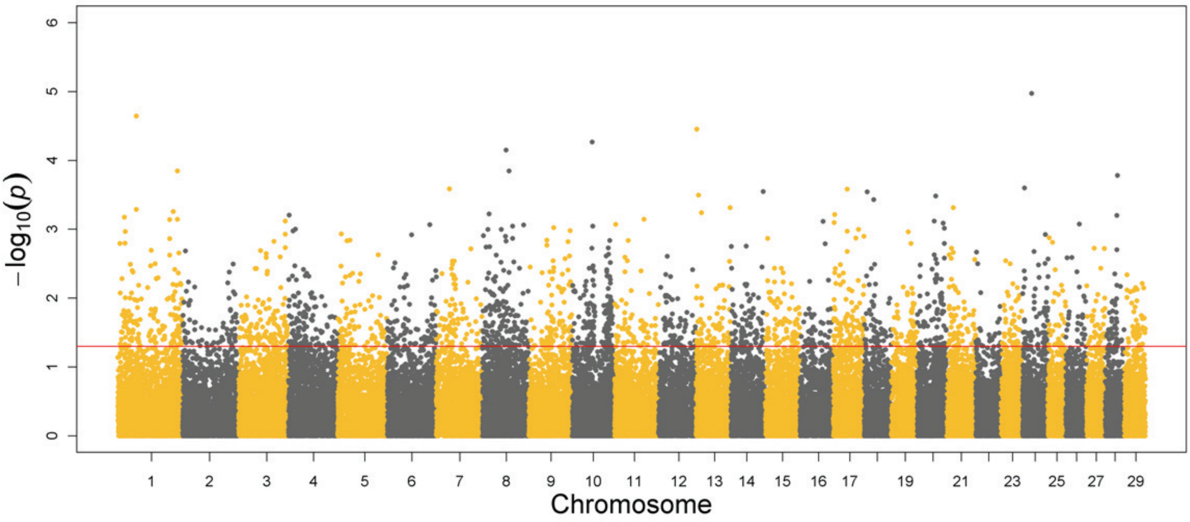

(D) Arginine

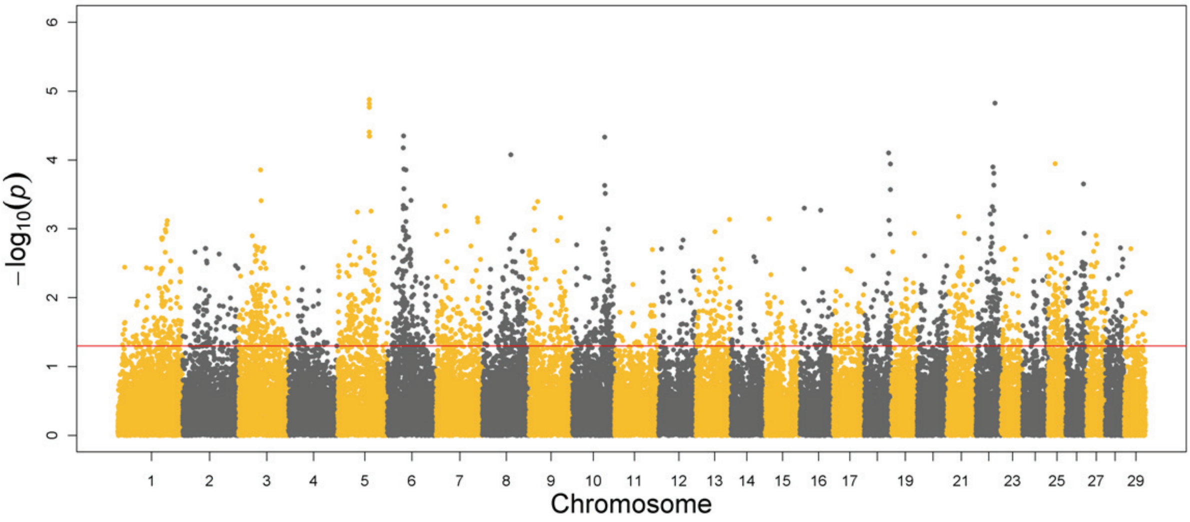

(E) Lysine

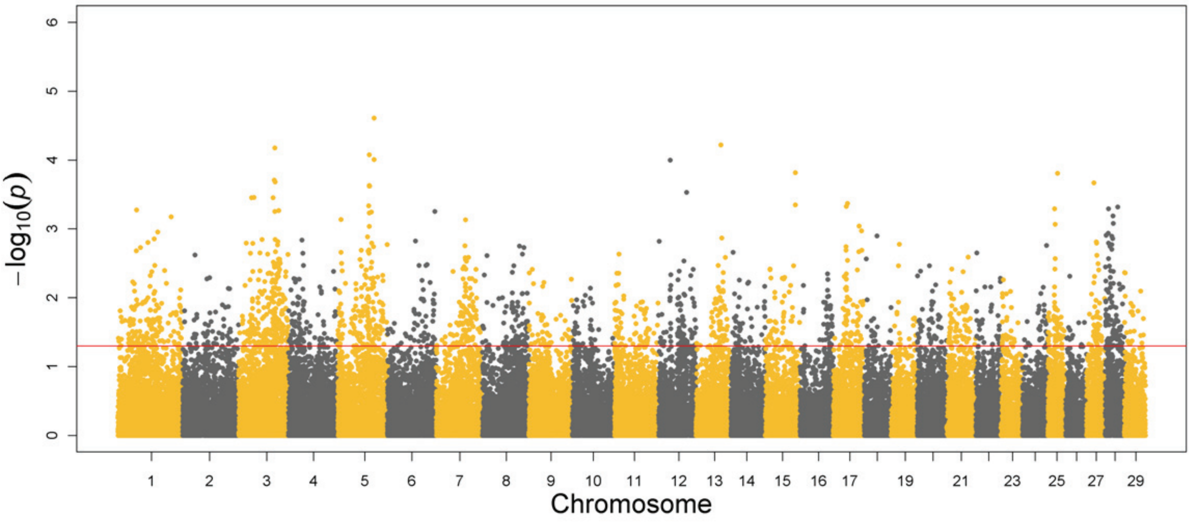

(F) C0

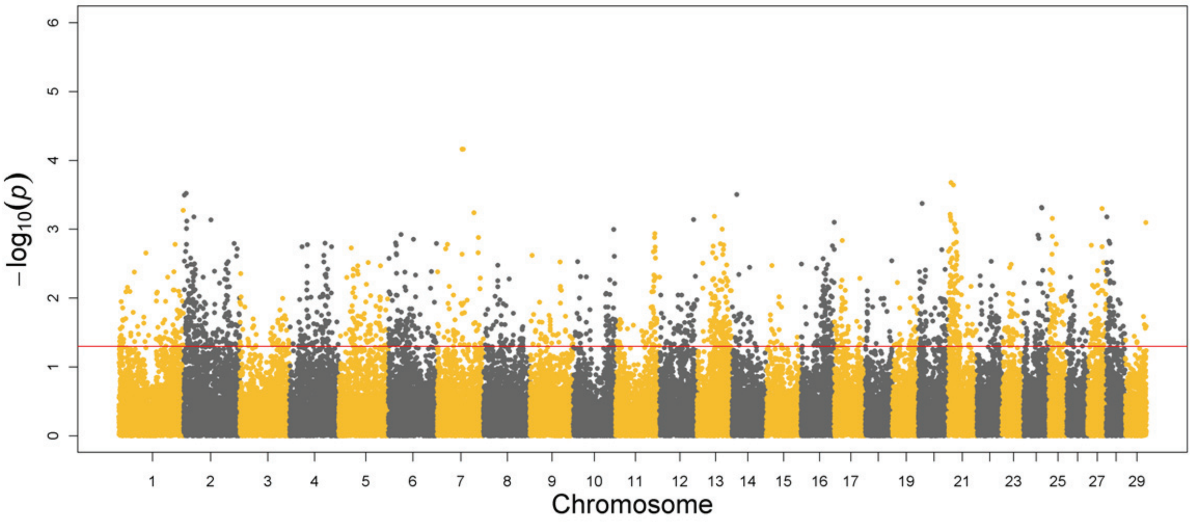

−log10(p)
0
1
2
3
4
5
6
1 2 3 4 5 6 7 8 9 10 11 12 13 14 15 16 17 19 21 23 25 27 29
Chromosome

(G) C2

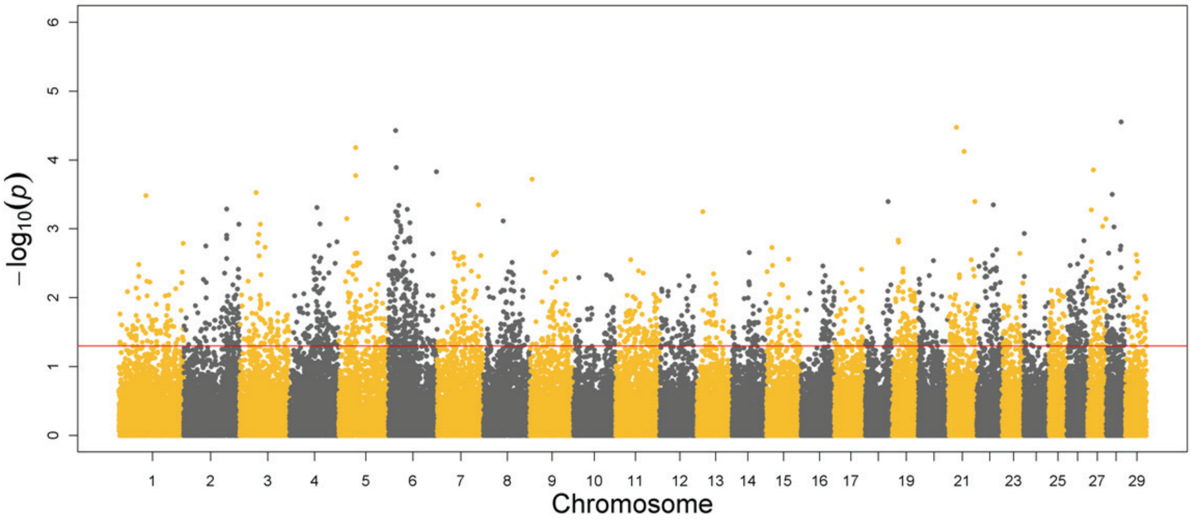

−log10(p)
0
1
2
3
4
5
6
1 2 3 4 5 6 7 8 9 10 11 12 13 14 15 16 17 19 21 23 25 27 29
Chromosome

(H) C5

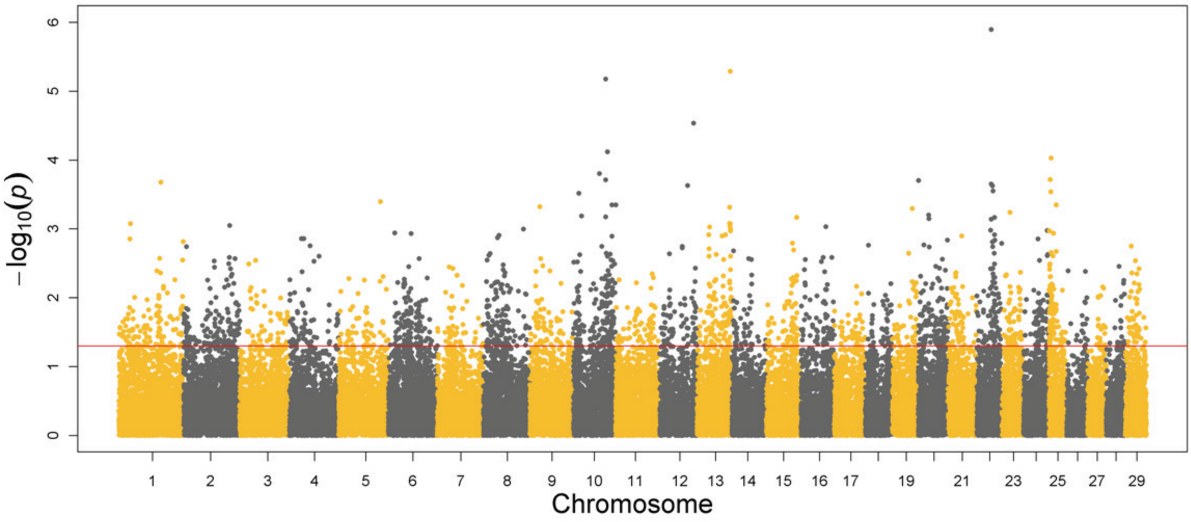

−log10(p)
0
1
2
3
4
5
6
1 2 3 4 5 6 7 8 9 10 11 12 13 14 15 16 17 19 21 23 25 27 29
Chromosome

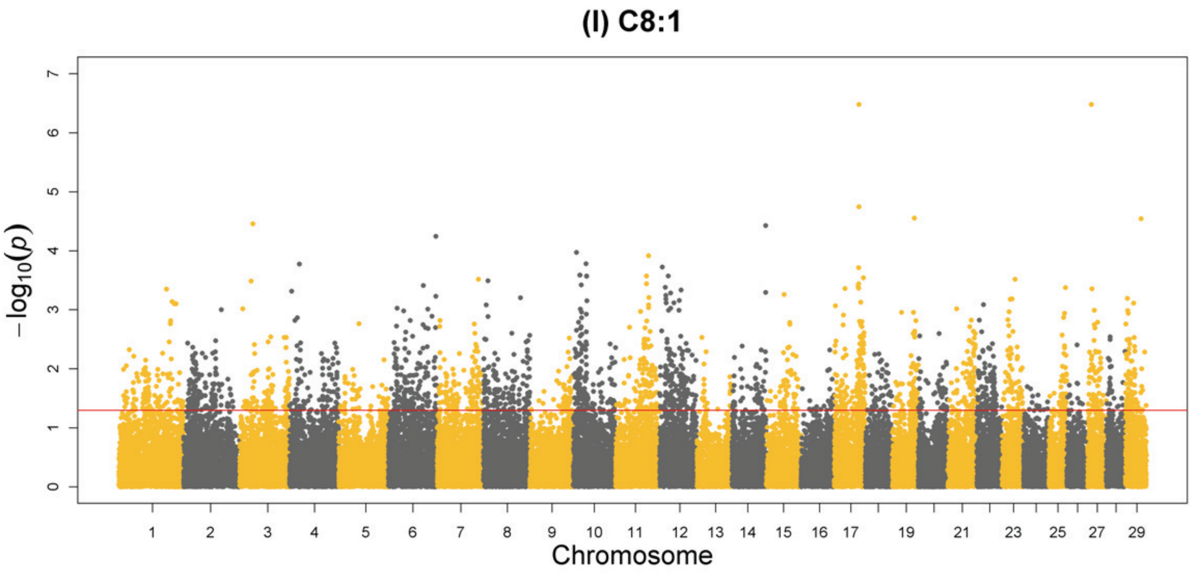
(I) C8:1
−log10(p)
0
1
2
3
4
5
6
7
1 2 3 4 5 6 7 8 9 10 11 12 13 14 15 16 17 19 21 23 25 27 29
Chromosome

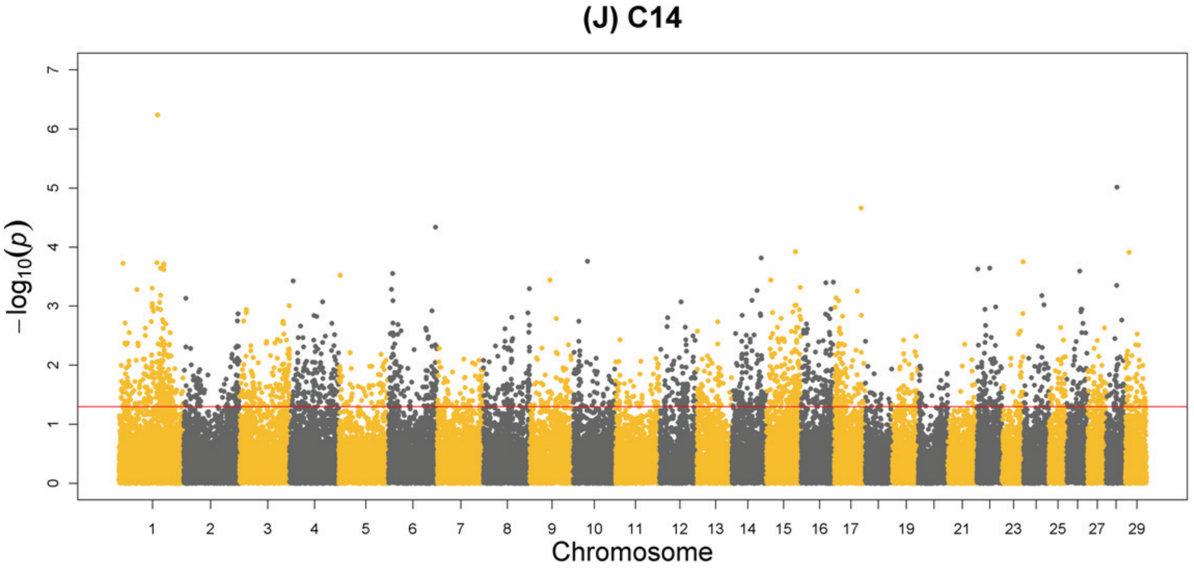
(J) C14
−log10(p)
0
1
2
3
4
5
6
7
1 2 3 4 5 6 7 8 9 10 11 12 13 14 15 16 17 19 21 23 25 27 29
Chromosome

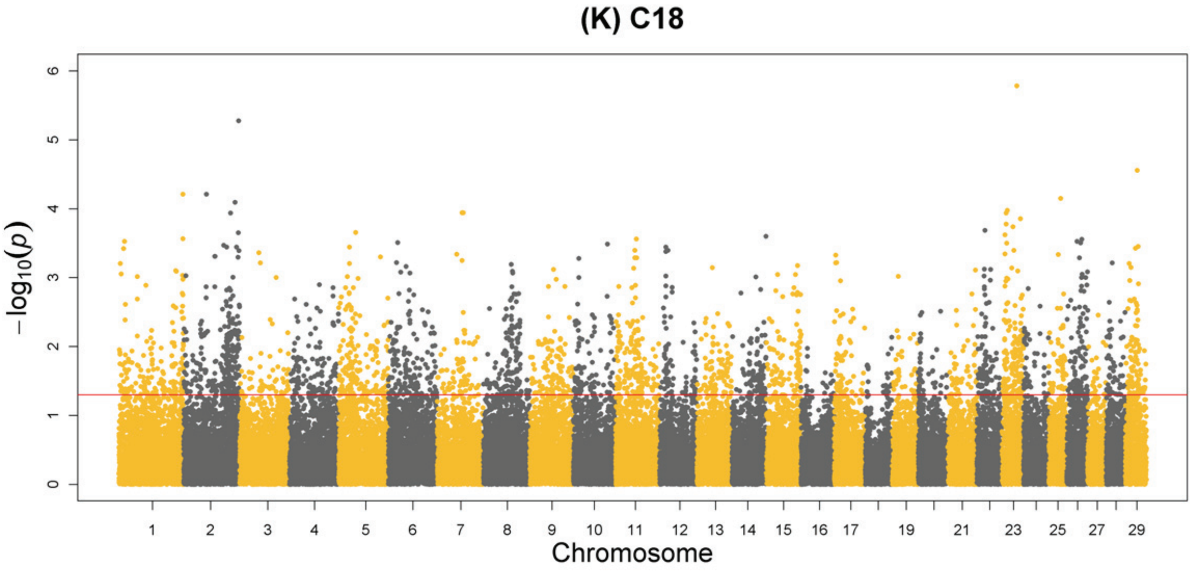
(K) C18
−log10(p)
0
1
2
3
4
5
6
1 2 3 4 5 6 7 8 9 10 11 12 13 14 15 16 17 19 21 23 25 27 29
Chromosome

(L) PC_aa_C32:0

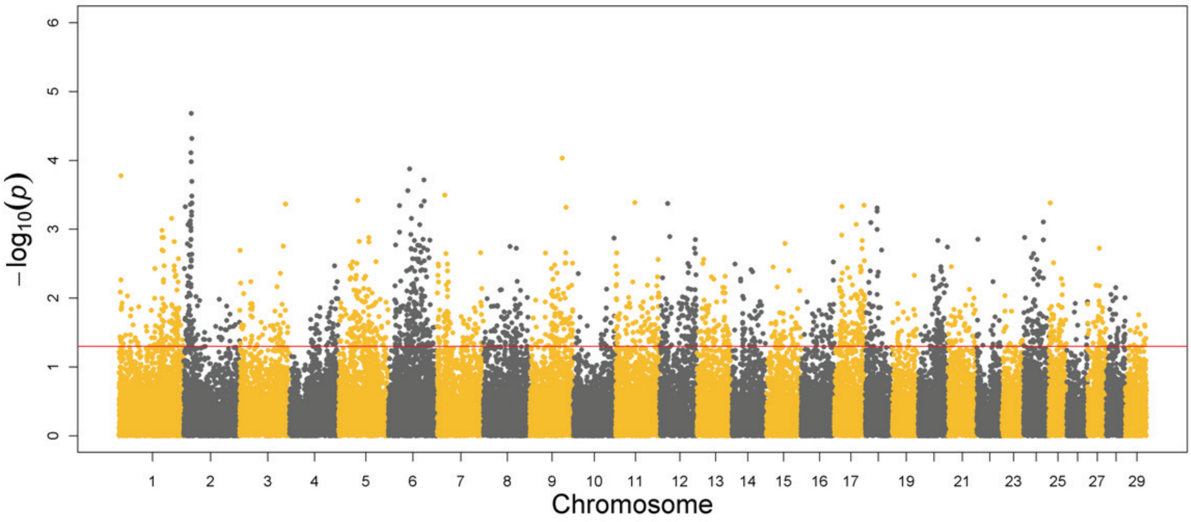

(M) PC_ae_C36:1

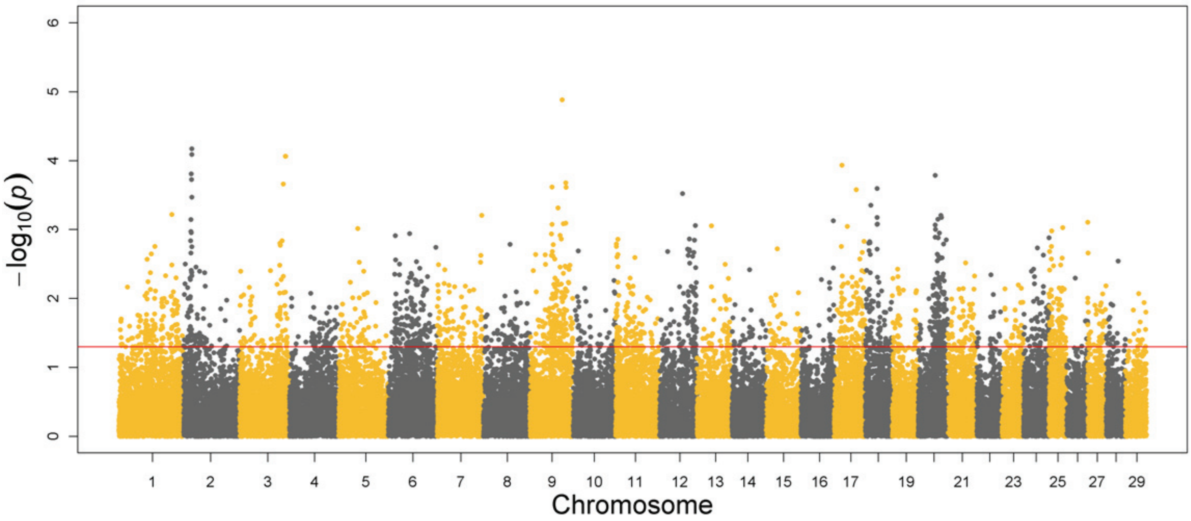

(N) SM_C20:2

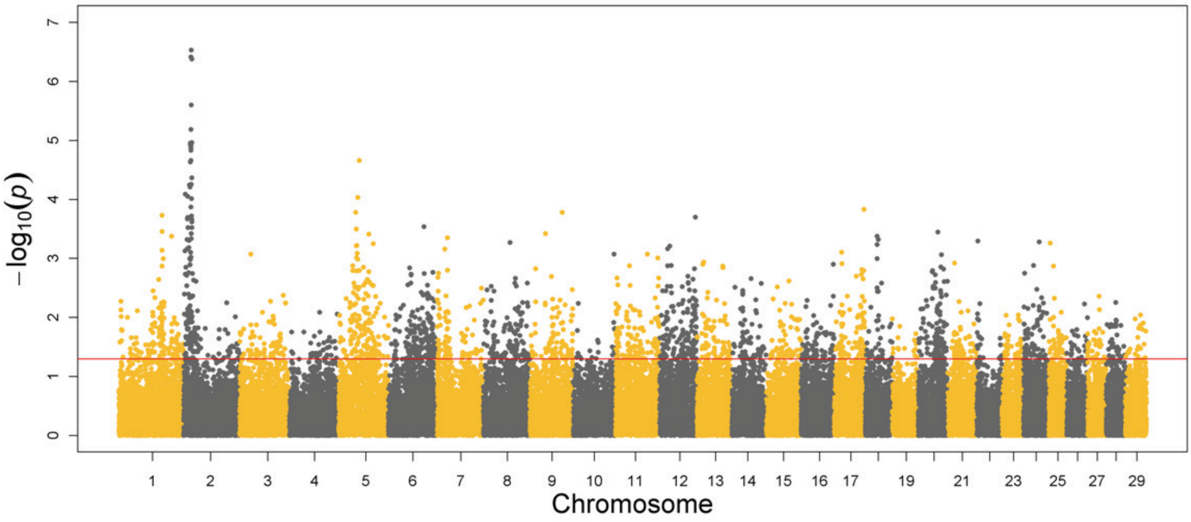

Supplement: S1 Fig — Manhattan plots resulting from the GWAS for (A) residual feed intake (RFI), (B) feed conversion rate (FCR), (C) daily energy intake (dEI), (D) arginine, (E) lysine, (F) free carnitine (C0), (G) acetylcarnitine (C2), (H) valerylcarnitine (C5), (I) suberylcarnitine (C8:1), (J) myristylcarnitine (C14), (K) stearoylcarnitine (C18); (L) diacylphosphatidylcholine C32:0 (PC_aa_C32:0), (M) acylethylphoshatidylcholine C36:1 (PC_ae_C36:1), (N) sphingomyelin C20:2 (SM_C20:2). Manhattan plots were plotted by the R package qqman [67]. (PDF) [file pone.0124574.s001.pdf]
